# Synergistic non‐covalent interactions enable high‐strength fluorescent supramolecular materials with water‐assisted self‐healing and remolding properties

**DOI:** 10.1002/smo2.70017

**Published:** 2025-08-26

**Authors:** Xiaoye Zhang, Haohui Wang, Pan Li, Hualin Tang, Tao Chen, Wei Lu

**Affiliations:** ^1^ State Key Laboratory of Advanced Marine Materials Ningbo Institute of Materials Technology and Engineering Chinese Academy of Sciences Ningbo China; ^2^ School of Chemical Sciences University of Chinese Academy of Sciences Beijing China; ^3^ Department of Orthopedic Trauma The First Affiliated Hospital of Ningbo University Ningbo China

**Keywords:** fluorescence, high strength, self‐healing, supramolecular materials, synergistic interactions

## Abstract

Supramolecular materials, characterized by dynamic reversibility and responsiveness to environmental stimuli, have found widespread applications in numerous fields. Unlike traditional materials, supramolecular materials that rely on non‐covalent interactions can allow spontaneous reorganization and self‐healing at room temperature. However, these materials typically exhibit low strength due to the weak bonding energies of non‐covalent interactions. This study presents the development of a high‐strength self‐healing supramolecular material that combines multiple interactions including ionic bonding, hydrogen bonding, and coordination bonding. The material, formed by the aggregation of the negatively charged picolinate‐grafted copolymer (PCM) with positively charged hyperbranched molecules (HP), is further enhanced by Eu^3+^ ion complexation. The resulting film exhibits a high modulus of 427 MPa, tensile strength of 10.5 MPa, and toughness of 14.7 MJ m^−3^. Meanwhile, the non‐covalent interaction of this supramolecular material endows it with a self‐healing efficiency of 92% within 24 h at room temperature, as well as multiple remolding properties. The incorporation of lanthanide ions also imparts tunable fluorescence. This study not only provides insights into the development of high‐strength self‐healing materials but also offers new possibilities for the functionalization of supramolecular materials.

## Graphical abstract

This work develops a supramolecular network utilizing multiple non‐covalent interactions including: ionic interactions, hydrogen bonding, and coordination bonding. The material not only has a high modulus, but also demonstrates good self‐healing properties, remolding properties, and multicolor tunability.

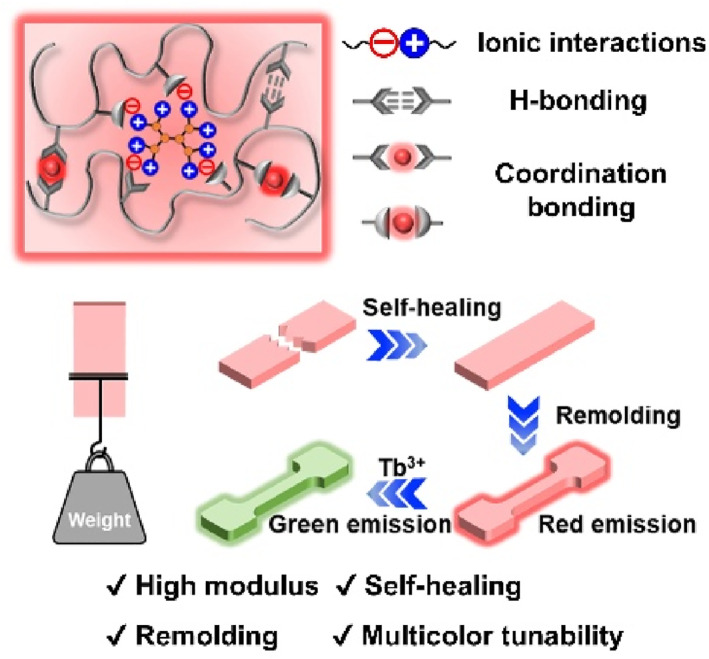

## 1 INTRODUCTION

Supramolecular materials are extensively utilized in fields such as self‐healing materials,[^1^, ^2^, ^3^, ^4^] biomedical applications,^[5]^ and other domains[^6^, ^7^] due to their dynamic reversibility and responsiveness to environmental stimuli.[^8^, ^9^] In contrast to traditional materials, the dynamics of supramolecular materials stem from their reversible bonding modes, which include non‐covalent interactions such as hydrogen bonding,[^10^, ^11^] metal‐ligand coordination,^[12]^ ion‐dipole interactions,^[13]^ host‐guest interactions,^[14]^ and electrostatic interactions,^[15]^ as well as dynamic covalent bonds,^[16]^ including disulfide bonds[^17^, ^18^, ^19^] and dynamic borate bonds.^[20]^ Among these, the breaking and reorganization of non‐covalent interactions can occur spontaneously at room temperature without external energy input, making them the most commonly employed bonding mechanism in self‐healing materials and smart gels.[^21^, ^22^, ^23^, ^24^, ^25^, ^26^] In contrast to traditional covalent bonds, the bonding energy of non‐covalent interactions is typically below 50 kJ/mol,^[8]^ which results in self‐healing materials that are generally soft and stretchable but still challenging to achieve high strength. Although the superposition of multiple weak interactions can form stronger bonds, their collective strength often remains insufficient to meet the required material stiffness during practical applications.

Numerous approaches have been employed to enhance the strength of supramolecular materials.[^27^, ^28^, ^29^] For instance, Zhang et al.^[30]^ developed a hydrogel with high stiffness and self‐healing properties by incorporating lithium montmorillonite nanosheets into an acrylamide network with shear‐induced orientation. The Young's modulus of this nano‐restricted hydrogel reached 50 MPa, which is 1–2 orders of magnitude higher than that of conventional hydrogels. Sun et al.^[31]^ developed self‐healing elastomers with tensile strengths of up to 40 MPa using polyurethane‐urea block copolymers. The urea group in the hard segment facilitates self‐healing through hydrogen bonding, while the polyether in the soft segment enhances the material's strength through the formation of crystalline regions. Wu et al.^[32]^ designed a glassy polymer material capable of autonomous self‐healing at room temperature by leveraging the high kinematic ability of the branched chains and terminal groups of hyperbranched polymers. This material benefited from the dense hydrogen bonding formed by the amide and amino groups, achieving a tensile strength of 18.5 MPa. However, this high‐strength glassy polymer material is inherently brittle, and its lack of toughness limits its adaptability in dynamic or complex stress environments. Therefore, the development of self‐healing supramolecular materials with high strength and toughness is essential.

**FIGURE 1:**
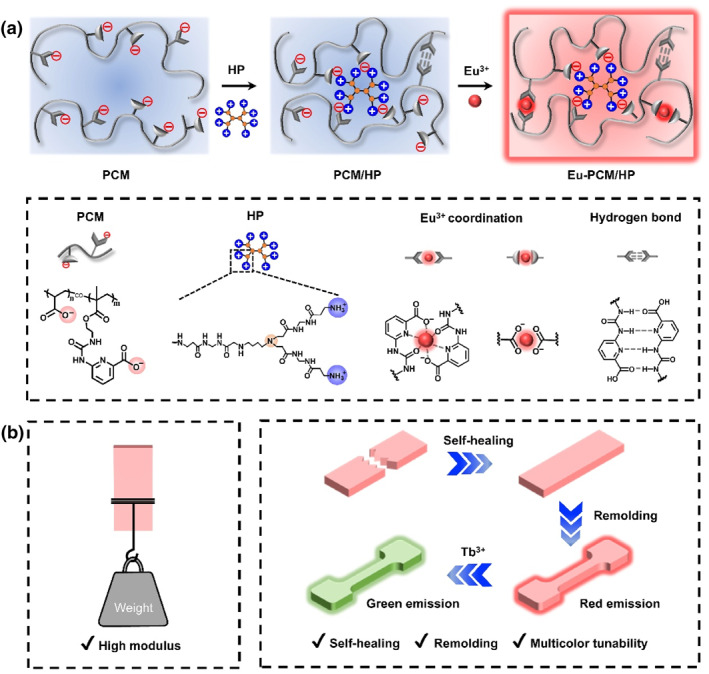
Fabrication of the Eu‐PCM/HP supramolecular films. (a) Scheme for the film materials preparation procedure. (b) Schematic illustration showing the properties of Eu‐PCM/HP including high modulus, self‐healing, remolding, and multicolor tunability.

In this study, a self‐healing supramolecular material with high modulus was developed by leveraging the synergistic enhancement of multiple interactions. The material is formed by the aggregation of a sodium acrylate and chelating ligand copolymer (PCM) and hyperbranched molecules (HP) through opposite charge‐induced aggregation, further strengthened by complexation with Eu^3+^ ions (Figure 1a). HP molecules, with their high mobility due to the peripheral branches and terminal functional groups, have been widely applied in the design of various self‐healing supramolecular materials, demonstrating excellent mechanical and self‐healing properties.[^15^, ^32^, ^33^] The supramolecular network remains in a glassy state at room temperature and contains multiple synergistic interactions, including hydrogen bonding, ionic interactions, and coordination bonding, resulting in high Young's modulus (427 MPa), tensile strength (10.5 MPa), and toughness (14.7 MJ m^−3^) (Figure 1b). Moreover, these dynamic and reversible interactions ensure that the material has a self‐healing efficiency of 92% within 24 h and multiple remodeling capabilities. The incorporation of lanthanide ions not only enhances the strength of the material system but also imparts tunable fluorescence properties. This study not only provides insights for constructing self‐healing materials with high mechanical strength but also offers opportunities for the functionalization of supramolecular materials.

## 2 RESULTS AND DISCUSSION

### 2.1 Design and preparation of Eu‐PCM/HP supramolecular materials

In order to construct supramolecular materials with high strength, two polymers with opposite charges were prepared (Figure 1a and Figure S1). The negatively charged polymer is a copolymer (PCM) consisting of sodium acrylate (AAs) and a specially designed organic ligand, 6‐(3‐(2‐(methacryloyloxy)ethyl)ureido)pyridinecarboxylate (6MUPA). The 6MUPA ligand is capable of forming coordination interactions with lanthanide ions, as well as tetraplex hydrogen‐bonding interactions between the two uncoordinated ligands.^[34]^ Another positively charged polymer is a hyperbranched molecule (HP) with highly mobile peripheral branches and amino‐terminal functional groups. The chemical structures of these two polymers were verified by ^1^H nuclear magnetic resonance spectroscopy (Figure S2). Then, the supramolecular films were prepared using a two‐step process, as illustrated in Figure S3. First, an aqueous solution of PCM was mixed with an aqueous solution of HP, resulting in the immediate formation of PCM/HP aggregates due to strong ionic interactions. In the second step, the resulting aggregates were immersed in a 0.01 M Eu(NO_3_)_3_ solution to facilitate coordination between the ligands and lanthanide ions, thereby further enhancing the supramolecular networks. The resulting wet precipitate is then transferred into a mold, where solvent evaporation leads to the formation of a dried supramolecular film, denoted as Eu‐PCM_*x*_/HP_*y*_. Here, *x*:*y* represents the mass ratio of PCM to HP in the formulation.

**FIGURE 2:**
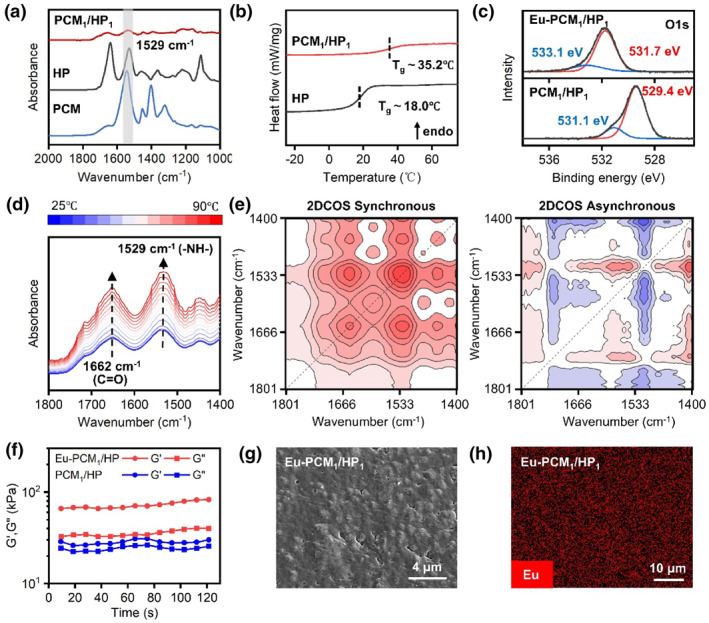
Characterization of the Eu‐PCM/HP supramolecular films. (a) FT‐IR spectra of PCM, HP, and PCM_1_/HP_1_. (b) DSC curves of HP and PCM_1_/HP_1_. (c) High‐resolution XPS fitting results for O1s spectra of PCM_1_/HP_1_ and Eu‐PCM_1_/HP_1_. (d) Temperature‐dependent FT‐IR spectra of PCM_1_/HP_1_ upon heating from 25°C to 90°C. (e) The corresponding 2DCOS synchronous spectra and asynchronous spectra, in which red and blue colors represent positive and negative intensity, respectively. (f) The rheology properties of Eu‐PCM_1_/HP_1_ and PCM_1_/HP_1_ at an oscillation frequency of 1 Hz. (g) SEM image of Eu‐PCM_1_/HP_1_ film. (h) EDS mapping of Eu in Eu‐ PCM_1_/HP_1_. 2DCOS, 2D correlation spectroscopy; DSC, differential scanning calorimetry; EDS, energy‐dispersive spectroscopy; FT‐IR, Fourier transform infrared spectroscopy; SEM, scanning electron microscope; XPS, X‐ray photoelectron spectroscopy.

Fourier transform infrared spectroscopy (FT‐IR) clearly revealed the complexation‐induced aggregation formation of PCM/HP (Figure 2a). The ‐NH‐ bending peak at ∼1529 cm^−1^ in HP became much weaker in the PCM/HP film, indicating strong ionic bonding between HP's amino groups and PCM's carboxyl groups. The differential scanning calorimetry curves in Figure 2b show that the glass transition temperature (*T*_g_) increased from 18.0°C in HP to 35.2°C in PCM_1_/HP_1_ after compounding with PCM due to the deterioration of polymer mobility. These results demonstrated the formation of supramolecular aggregation networks driven by ionic interactions. To better understand the ionic complexation between PCM and HP, PCM/HP films were analyzed by using variable‐temperature FT‐IR spectroscopy. As shown in Figure 2d, the ‐C=O and ‐NH‐ peaks did not shift with temperature, but their intensities increased noticeably. This suggests that the ionic bonds between COO^−^ and protonated ‐NH‐ groups weaken as the temperature rises from 25°C to 90°C. 2D correlation spectroscopy (2DCOS) analysis was further conducted to reveal the microdynamic mechanism of this dynamic crosslinking system in the heating process (Figure 2e). In the synchronous map, two autopeaks at 1662 and 1529 cm^−1^ can be clearly identified, corresponding to the vibration peaks of ‐C=O and ‐NH‐ moieties. The ‐C=O groups responded prior to the ‐NH‐ ones, possibly owing the hydrogen bond network formed by many carbonyl groups in the PCM molecule is enough unstable. After that, Eu^3+^ ions were further introduced to enhance the supramolecular network and bring fluorescence properties. X‐ray photoelectron spectroscopy (XPS) was used to confirm the successful incorporation of Eu^3+^ ions. Upon complexation, the O1s binding energies increase from 529.4 and 531.1 eV to 531.7 and 533.1 eV, respectively, indicating stronger and more stable interactions between Eu^3+^ ions and oxygen atoms (Figure 2c). This is further supported by the presence of the Eu4d peak in the XPS spectra (Figure S4). The FT‐IR spectra, both before and after Eu^3+^ complexation, are also provided in Figure S5. Rheological measurements showed that both the storage modulus (G′) and loss modulus (G″) of Eu‐PCM_1_/HP_1_ were higher than those of PCM_1_/HP_1_ as a result of the coordination cross‐linking of lanthanide ions (Figure 2f). Following solvent evaporation, a dry brown transparent supramolecular film (Eu‐PCM/HP) was obtained. In the visible region, the transmittance of Eu‐PCM_1_/HP_1_ increased from 20% at 400 nm to approximately 80% at 600 nm and remained above 80% from 600 to 800 nm (Figure S6). Scanning electron microscope (SEM) image and energy‐dispersive spectroscopy (EDS) demonstrated the surface micromorphology of the films and the homogeneous distribution of Eu elements in the material (Figure 2g,h).

### 2.2 Mechanical property of Eu‐PCM/HP film

**FIGURE 3:**
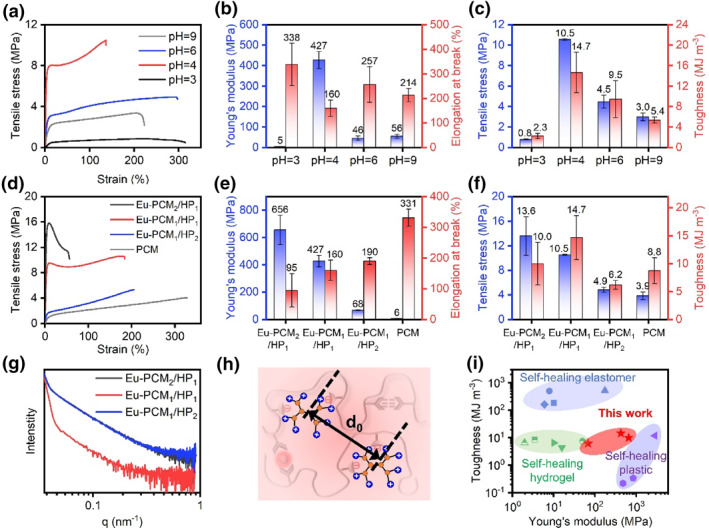
Mechanical property of Eu‐PCM/HP film. (a) Tensile stress‐strain curves of supramolecular films prepared from PCM solutions with different pH. (b, c) Young's modulus, elongation at break, tensile strength, and toughness of these film samples. Error bars represent the SD based on five independent replicates. (d) Tensile stress‐strain curves of supramolecular film with different component ratios. (e, f) Young's modulus, elongation at break, tensile strength, and toughness of these film samples. Error bars represent the SD based on five independent replicates. (g) SAXS spectrum for supramolecular film with different component ratios. (h) Illustrations showing the periodic structural period d_0_ in the Eu‐PCM/HP film. (i) Materials property charts of toughness versus Young's modulus of various self‐healing materials, including self‐healing elastomers, self‐healing hydrogels, and self‐healing plastics. SAXS, small‐angle X‐ray scattering; SD, standard deviation.

After demonstrating the successful preparation of Eu‐PCM/HP supramolecular materials, their mechanical properties were further probed. The glass transition temperature of the Eu‐PCM_1_/HP_1_ supramolecular film determines its mechanical properties of high modulus and high strength at room temperature (Figure 2d). Interestingly, the pH of the PCM aqueous solution in preparation step 1 significantly affects the mechanical properties of the materials including Young's modulus, elongation at break, tensile strength, and toughness (Figure 3a–c). This is because the pH influences the degree of ionization and charge density of the PCM solution (Figure S7). When the PCM solution is alkaline (pH > 8), the copolymer becomes more ionized, leading to the formation of a significant number of negatively charged carboxylate groups. The osmotic pressure effect of these carboxylate ions, along with their hydration capacity, leads to the adsorption of water molecules into the prepared supramolecular network, making them difficult to remove through solvent evaporation. Consequently, the Young's modulus and tensile strength of the resulting material are only 56 and 3.0 MPa, respectively. When the solution of PCM is acidic (pH ≤ 3), the ionization degree of PCM molecules is low, and there is not enough negative charge to drive the formation of supramolecular network with high crosslink density. As a result, the obtained materials were soft and stretchable with elongation at break exceeding 300%, but the Young's modulus and tensile strength were only 5 and 0.8 MPa. By adjusting and optimizing the solution environment of PCM, it was found that excellent mechanical properties can be achieved when the pH is between 4 and 6. Notably, at pH = 4, the prepared Eu‐PCM_1_/HP_1_ supramolecular material exhibited a Young's modulus of 427 MPa and a tensile strength of 10.5 MPa, surpassing the mechanical strength of many supramolecular materials. Additionally, the film demonstrated an elongation at break of 160% and a toughness of 14.7 MJ m^−3^, effectively avoiding the brittle fracture typically seen in glassy polymer materials. This enhanced ductility may be attributed to trace amounts of solvent retained within the material.

In addition to the solution environment, the composition ratio of the two polymers affects the crosslinking strength of the supramolecular materials. The successful preparation of materials with different composition ratios named Eu‐PCM_1_/HP_2_ and Eu‐PCM_2_/HP_1_ was demonstrated by FT‐IR, SEM, and EDS, respectively (Figures S8 and S9). The incorporation of positively charged HP molecules significantly enhanced the strength of the material compared to the pure PCM film (Figure 2d–f). In contrast, Eu‐PCM_1_/HP_2_ shows only a modest improvement in mechanical strength due to insufficient cross‐linking. The highest Young's modulus and tensile strength are observed in Eu‐PCM_2_/HP_1_; however, this comes at the cost of reduced elongation at break (<100%) and low toughness (10.0 MJ m^−3^). Overall, when the mass ratio of PCM to HP is 1:1, the material exhibits optimal performance, achieving both high mechanical strength and toughness. Remarkably, the supramolecular film was able to lift weights more than 4500 times its own weight without deformation (Figure S10). Similarly, the Eu‐PCM_1_/HP_2_ and Eu‐PCM_2_/HP_1_ films were able to lift weights of 500 g and 1 kg, respectively (Figure S10). To explore the factors contributing to the differences in material properties, small‐angle X‐ray scattering was carried out (Figure 3g). The periodic sizes of the aggregate structures in Eu‐PCM_1_/HP_1_ are only 34 nm, compared to approximately 64 nm in both Eu‐PCM_2_/HP_1_ and Eu‐PCM_1_/HP_2_ (Figure 3h). This indicates that the aggregation structures in the Eu‐PCM_1_/HP_1_ film are more uniformly distributed, effectively transferring and distributing stress, which results in better mechanical strength and toughness. The high mechanical properties of this supramolecular material are attributed to the unique design of its structure. In the supramolecular crosslinked network, the polymer chains are in a glassy state, contributing to high modulus and strength. Furthermore, the system is enriched with a variety of non‐covalent interactions, including ionic, ligand, and hydrogen bonding, which further enhance the crosslinking strength. Although individual non‐covalent bonds may have low strength, their synergistic effect significantly enhances the overall mechanical properties of the material. The combination of high Young's modulus and toughness of this supramolecular material makes it a leader among a number of self‐healing materials, including self‐healing elastomers,[^31^, ^35^, ^36^] self‐healing hydrogels,[^30^, ^37^, ^38^, ^39^, ^40^] and self‐healing plastics[^32^, ^41^, ^42^] (Figure 3i).

### 2.3 Self‐healing and remolding properties of Eu‐PCM_1_/HP_1_ film

**FIGURE 4:**
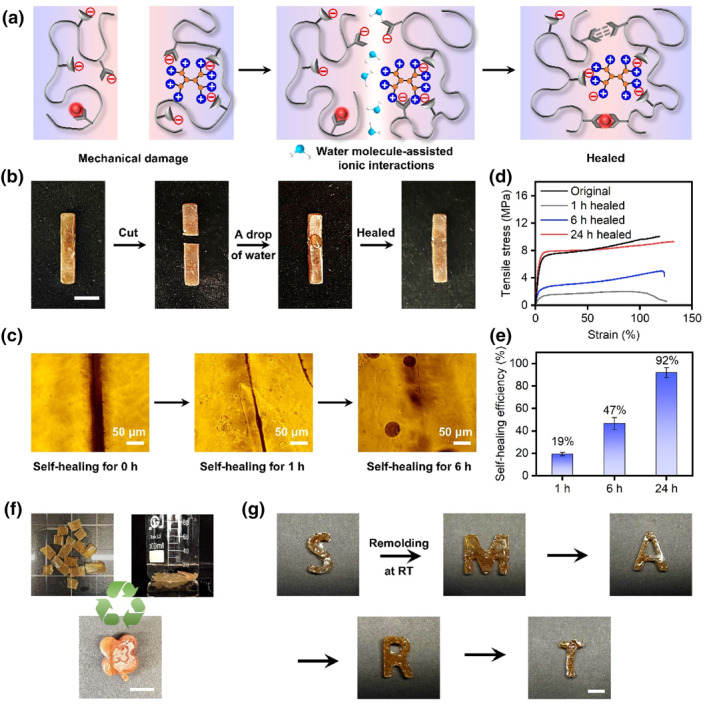
Self‐healing and remolding properties of Eu‐PCM_1_/HP_1_ film. (a) Schematic illustration of the self‐healing process of supramolecular materials facilitated by water molecules. (b) Photographs showing the self‐healing process of Eu‐PCM_1_/HP_1_ film. (c) Microscopic images showing the self‐healing process. (d) Tensile stress‐strain curves of the Eu‐PCM_1_/HP_1_ film and the self‐healed Eu‐PCM_1_/HP_1_ film at various healing durations. (e) Self‐healing efficiency of the Eu‐PCM_1_/HP_1_ film at different healing times. Error bars represent the SD based on three independent replicates. (f) Photographs showing the recycling process of Eu‐PCM_1_/HP_1_ film. (g) Photos showing the shape remolding ability of the Eu‐PCM_1_/HP_1_ sample from the first time to the fifth time. Scale bar for (b, f, and g) is 1 cm. SD, standard deviation.

In addition to its excellent mechanical strength, the supramolecular network structure endows the material with intrinsic self‐healing properties. Notably, this self‐healing ability requires the presence of water molecules; films in a completely dry state do not exhibit self‐healing behavior. This is primarily because, the polymer chains are immobilized in a frozen state in the absence of water. The introduction of water molecules restores the mobility of the polymer chains and promotes ionization, leading to the generation of additional oppositely charged species (Figure 4a). When the supramolecular material is mechanically damaged, the two parts are brought back into contact and a drop of water is added to the interface. As a result, the two parts will quickly heal again (Figure 4b). Micrographs of the supramolecular film visually demonstrate the healing process. As shown in Figure 4c and Figure S11, the scar was lighter after 1 h of healing and nearly disappeared after 6 h of healing. Tensile testing provided a clearer picture of the material's self‐healing capability. As shown in the stress‐strain curves in Figure 4d, the material recovered 20% of its original strength and full stretchability after 1 h of healing. Surprisingly, after 24 h of healing, the curve nearly overlaps with that of the original sample. As healing time increases, the self‐healing efficiency steadily improves, reaching 92% at 24 h (Figure 4e). Another key property of supramolecular materials with multiple interactions is their ability to undergo remolding (Figure 4f). When the material is cut into small pieces and immersed in water, the ionization of the abundant functional groups on the polymer induces the reorganization of the interactions. After the material has been submerged in water for more than 48 h, the film becomes soft and can be plasticized and remolded into a variety of shapes several times through different molds (Figure 4g). The remolding ability enhances the processability of Eu‐PCM/HP materials, expanding their potential applications and broadening the range of scenarios in which they can be used.

### 2.4 Multicolor tunability of the supramolecular films

**FIGURE 5:**
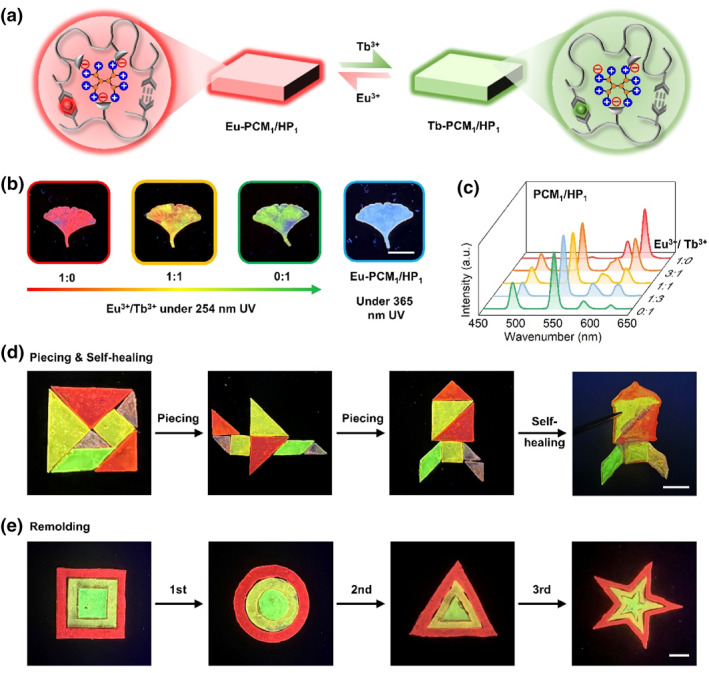
Fluorescence performance of the supramolecular PCM_1_/HP_1_ films. (a) Schematic illustration of Eu‐PCM_1_/HP_1_ and Tb‐PCM_1_/HP_1_ with red or green fluorescence. (b) Photographs of the PCM_1_/HP_1_ film treated with mixed Eu^3+^/Tb^3+^ ions taken under a 254 nm UV lamp. The right panel shows a photograph of the Eu‐PCM_1_/HP_1_ film with blue fluorescence under a 365 nm UV lamp. (c) Fluorescence spectra of the multicolor fluorescent supramolecular films with different Eu/Tb molar ratios. (d) Photographs showing the fluorescence and self‐healing capabilities of the supramolecular films taken under a 254 nm UV lamp. (e) Photographs showing the fluorescence and remolding capabilities of the supramolecular films taken under a 254 nm UV lamp. Scale bar for (b, d, and e) is 1 cm.

The coordination of lanthanide ions endowed the supramolecular material with tunable multicolor fluorescence, as shown in Figure 5a. The Eu‐PCM_1_/HP_1_ film exhibited bright red fluorescence under 254 nm UV illumination. Replacing Eu(NO_3_)_3_ with Tb(NO_3_)_3_ solution in preparation step 2 resulted in a supramolecular film with green fluorescence. XPS (Figure S4), SEM, and EDS (Figure S12) confirm the successful preparation of the Tb‐PCM_1_/HP_1_ film. Therefore, multicolor fluorescent films were obtained by immersing in mixed solutions of Eu^3+^ and Tb^3+^, as shown in Figure 5b. Fluorescence spectroscopy investigation revealed that the intensity changes of the green band and red band as the molar ratio of Eu^3+^/Tb^3+^ varies (Figure 5c). Additionally, the specially designed pyridine carboxylic acid ligand (6MUPA) in PCM emits blue fluorescence under 365 nm UV light (right panel in Figure 5b), which can also be seen in the two‐dimensional fluorescence spectra (Figure S13). Supramolecular materials with different component ratios exhibit similar fluorescence properties (Figure S14). Using the fluorescence properties of the supramolecular materials, the self‐healing behavior of the supramolecular film can be observed more intuitively. After the PCM_1_/HP_1_ film was mechanically cut into two parts and treated with Eu^3+^ and Tb^3+^ ions, the two parts showed red and green fluorescence under UV light, respectively. When the two parts were brought back together and a small amount of deionized water was added dropwise at the interface, the two parts healed into a single unit after 6 h (Figure S15). By combining the fluorescence and healing properties of this supramolecular material, the square film was cut into puzzle shapes and treated with different lanthanide ions separately. After stitching and arranging the pieces together, they were formed into “airplane” or “rocket” shapes. Through water‐assisted healing, the fragments could be transformed into a complete rocket‐shaped material with different fluorescent color regions, as shown in Figure 5d. In addition, the supramolecular film with multicolor fluorescence can be remolded multiple times into different shapes (Figure 5e). In conclusion, the tunable fluorescence properties of this supramolecular material offer new possibilities for the development of colorful, patterned materials.

## 3 CONCLUSIONS

In this study, a self‐healing supramolecular material with high modulus and strength was successfully developed by leveraging the synergistic effects of multiple interactions including ionic, hydrogen bonding, and coordination bonding. The material was prepared by combining a sodium acrylate and chelating ligand copolymer (PCM) with hyperbranched molecules (HP), which aggregate through opposite charge interactions, and further strengthened through complexation with Eu^3+^ ions. The resulting supramolecular film exhibited excellent mechanical properties, including a modulus of 427 MPa, tensile strength of 10.5 MPa, and toughness of 14.7 MJ m^−3^. Additionally, the material maintained its self‐healing and remolding ability, which was attributed to the dynamic and reversible interactions within the polymer network. The incorporation of lanthanide ions also imparted tunable fluorescence properties, adding a functional dimension to the material that could be utilized in various applications.

The material's ability to heal itself at room temperature without external energy input underscores its potential for use in practical, real‐world applications. Furthermore, the incorporation of lanthanide ions enhances both the mechanical strength and the functionality of the material, offering new opportunities for the design of multifunctional supramolecular materials. This work highlights the importance of synergizing multiple non‐covalent interactions to overcome the inherent weaknesses of individual interactions, providing a pathway toward the development of advanced materials that combine high mechanical performance with self‐healing and functional capabilities.

## CONFLICT OF INTEREST STATEMENT

The authors declare no conflicts of interest.

## ETHICS STATEMENT

No animal or human experiments were involved in this study.

## Supporting information

Supporting Information S1

## Data Availability

The data that support the findings of this study are available from the corresponding author upon reasonable request.
