# Supplementary material for: Synergistic non‐covalent interactions enable high‐strength fluorescent supramolecular materials with water‐assisted self‐healing and remolding properties
Source: Smart Mol. 2025 Aug 26;3(3):e70017. doi: 10.1002/smo2.70017 (PMC12483129; doi:10.1002/smo2.70017)
Supplement: Supplementary file 1 — Supporting Information S1 [file SMO2-3-e70017-s001.docx]

Supporting Information

**Synergistic Non-Covalent Interactions Enable High-strength Fluorescent Supramolecular Materials with Water-Assisted Self-Healing and Remolding Properties**

*Xiaoye Zhang†, Haohui Wang†, Pan Li, Hualin Tang*, Tao Chen*, and Wei Lu**

Table of content

Materials and methods

Figure S1-S15.

**Materials and methods**

***Materials:*** 6-Amino-pyridine-2-carboxylic acid (98%), 2-isocyanatoethyl methacrylate (98%), and triethylamine (99.5%, Extra Dry) were supplied by Energy Chemical. Sodium acrylate (AAs, 98.0%), ammonium persulfate (APS, 98%), and sodium hydroxide (NaOH, 97%) were purchased from Aladdin Co. Ltd. N,N'-Methylenebis(acrylamide) (MBA, 99%) and 1,4-Diaminobutane (BDA, 98%) were purchased from Macklin Co. Ltd. Eu(NO_3_)_3_·6H_2_O (99.9%), and Tb(NO_3_)_3_·5H_2_O (99.9%) were obtained from Meryer Co. Ltd. Methanol, acetone, and HCl (37%) were obtained from Sinopharm Chemical Reagent Co. Ltd. All chemicals were used as received without further purification. The ligand monomer of 6-(3-(2-(methacryloyloxy)ethyl)ureido)picolinic acid (6MUPA) was synthesized according to previously reported methods^1^.

***Synthesis of the PCM:*** The linear copolymer poly(sodium acrylate-co-sodium 6-(3-(2-(methacryloyloxy)ethyl)ureido)pyridinecarboxylate) (PCM) was synthesized via thermally initiated radical polymerization (**Figure S1**). The molar ratio of the chelating ligand to acrylamide was maintained at 1:99, and the product was characterized by nuclear magnetic resonance (NMR), as shown in **Figure S2**. To begin, an aqueous solution of Na6MUPA was prepared by dissolving 400 mg of 6MUPA (ligand monomer) and 55 mg of NaOH in 10 mL of deionized water. Separately, 12.66 g of AAs (acrylate monomer) was dissolved in 50 mL of deionized water, after which the Na6MUPA solution was added. Subsequently, 130 mg of ammonium persulfate (APS) was introduced as an initiator, and the mixture was polymerized at 70 °C for 5 hours. The resulting reaction mixture was then poured into 500 mL of methanol to precipitate the polymer. The precipitate was collected by filtration and washed three times with methanol. Finally, the polymer product was dried in a vacuum oven to obtain the purified copolymer.

***Synthesis of the HP:*** The hyperbranched polymers (HP) were prepared via a one-pot synthesis based on a Michael addition reaction between MBA and BDA according to previously reported methods^2^ (**Figure S1**). The structural characterization of the HP is shown in **Figure S2**. A representative synthesis procedure is outlined as follows: MBA (12.33 g, 0.08 mol) was introduced into a round-bottom flask equipped with a magnetic stirrer, containing a solvent mixture of 60 mL methanol and 30 mL deionized water. Separately, BDA (8 g, 0.09 mol) was dissolved in 20 mL methanol and 10 mL deionized water, and the resulting solution was directly transferred into the flask. The reaction was allowed to proceed under continuous stirring at 30 °C for 48 hours. Upon completion, the reaction mixture was poured into 1000 mL of acetone at room temperature to induce polymer precipitation. The crude product was collected and washed with acetone three times to remove impurities, followed by drying in a vacuum oven at 50 °C for 48 hours to yield the final solid polymer.

***Preparation of Eu-PCM/HP films:*** The supramolecular film was fabricated through a two-step process. First, each of the two polymers was individually dissolved in deionized water, and the pH of each solution was adjusted to a specific value. Upon mixing the two solutions, a precipitate was formed. This precipitate was then transferred into Eu(NO₃)₃ solution, and after soaking, the solvent was evaporated to obtain the final polymer film. Taking the Eu-PCM_1_/HP_1_ film as an example, the preparation procedure is summarized as follows: 2 g of PCM was dissolved in 10 mL of deionized water and titrated with 1 mol/L HCl to adjust the pH to 4.0 ± 0.1. Similarly, 2 g of HP was dissolved in 10 mL of deionized water and titrated to a pH of 7.0 ± 0.1. Upon combining the two solutions, a precipitate formed and was left to stand undisturbed for 12 hours. The resulting precipitate was then immersed in a 0.01 M Eu³⁺ solution and allowed to soak for 10 minutes. Subsequently, the precipitate was transferred into a polytetrafluoroethylene (PTFE) mold (40 mm in diameter), and the solvent was evaporated over a period of at least 72 hours to produce the dry Eu-PCM_1_/HP_1_ supramolecular film. The resulting samples were then ready for subsequent mechanical property testing.

***Characterization:*** Mechanical properties of the samples were characterized by a mechanical testing machine (Zwick/Roell Z1.0, Germany) using an extension rate of 100 mm/min at room temperature. For measurement of Young's modulus, elongation at break, tensile strength, and toughness, rectangle samples (5 mm in width, 1mm in thickness) were used. These mechanical properties were determined for each set of samples as the mean value of at least 5 samples. For self-healing efficiency measurements, at least three average values should be taken for each group of samples. The surface morphologies of the samples were examined using field-emission scanning electron microscopy (FE-SEM, Hitachi S-4800, Japan). Proton nuclear magnetic resonance (^1^H NMR) spectra were acquired on a Bruker Avance III 400 MHz spectrometer (USA), with PCM and HP dissolved in D₂O. Fourier transform infrared (FT-IR) spectroscopy was conducted using a Thermo Fisher Scientific NICOLET 6700 spectrometer (USA) with a resolution of 2.0 cm^−1^ in variable temperature infrared testing and 0.5 cm^−1^ in other infrared testing, respectively. The storage moduli G’ and loss moduli G″ of the samples were collected at a strain of 0.1% at a constant frequency of 1 Hz on a rotational rheometer (TA HR-3, USA) and parallel plate setup. X-ray photoelectron spectroscopy (XPS) measurements were carried out using an AXIS Ultra spectrometer (Japan). Optical transmittance spectra were recorded with a PerkinElmer LAMBDA 950 UV/Vis/NIR spectrophotometer (USA). Small-angle X-ray scattering (SAXS) measurements were performed on a Xeuss 3.0 UHR system (Xenocs, France) with an X-ray wavelength of λ = 1.54 Å (Cu K_α_). Samples were mounted on a solid sample stage with a sample-to-detector distance of 2000 mm and exposed to X-rays for 10 minutes. Sample photographs were captured using a smartphone camera, while fluorescence images were recorded under UV illumination (UVL-28, 8 W, 254 nm) using the same device. Differential scanning calorimetry (DSC) analyses were conducted using a Mettler Instruments Q200 calorimeter under a nitrogen flow of 50 mL min⁻¹. Approximately 5 mg of each sample was heated from –100 °C to 100 °C at a rate of 10 °C·min⁻¹. The second heating curves were used to calculate the glass transition temperatures. Steady-state fluorescence spectra of the hydrogels were measured using a Horiba F4700 fluorescence spectrophotometer (Japan).

**Figure S1.**

**
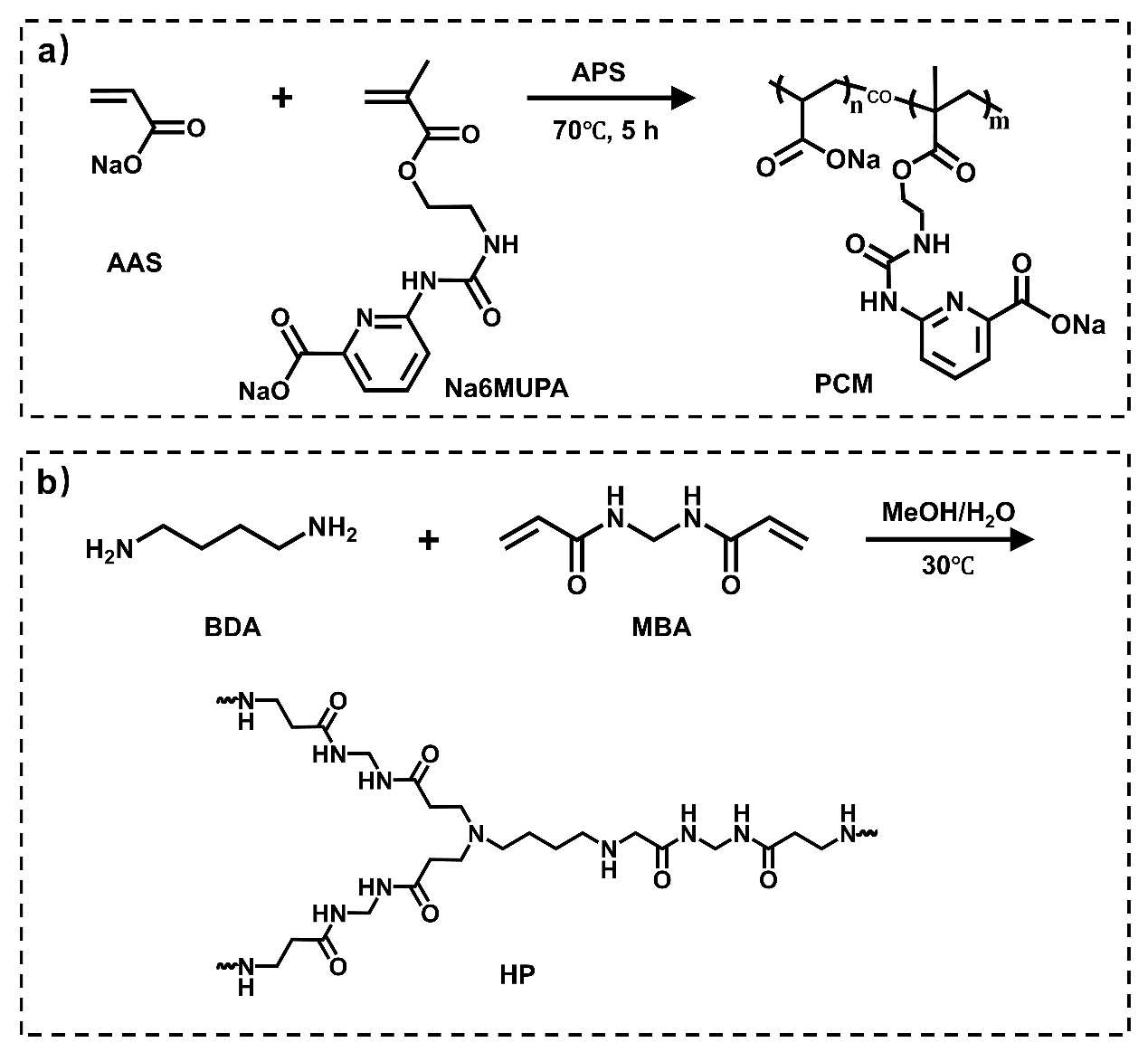
**

**Figure S1.** Preparation of PCM and HP. (a) Schematic diagram of synthetic reaction of PCM. (b) Schematic diagram of synthetic reaction of HP.

**Figure S2.**


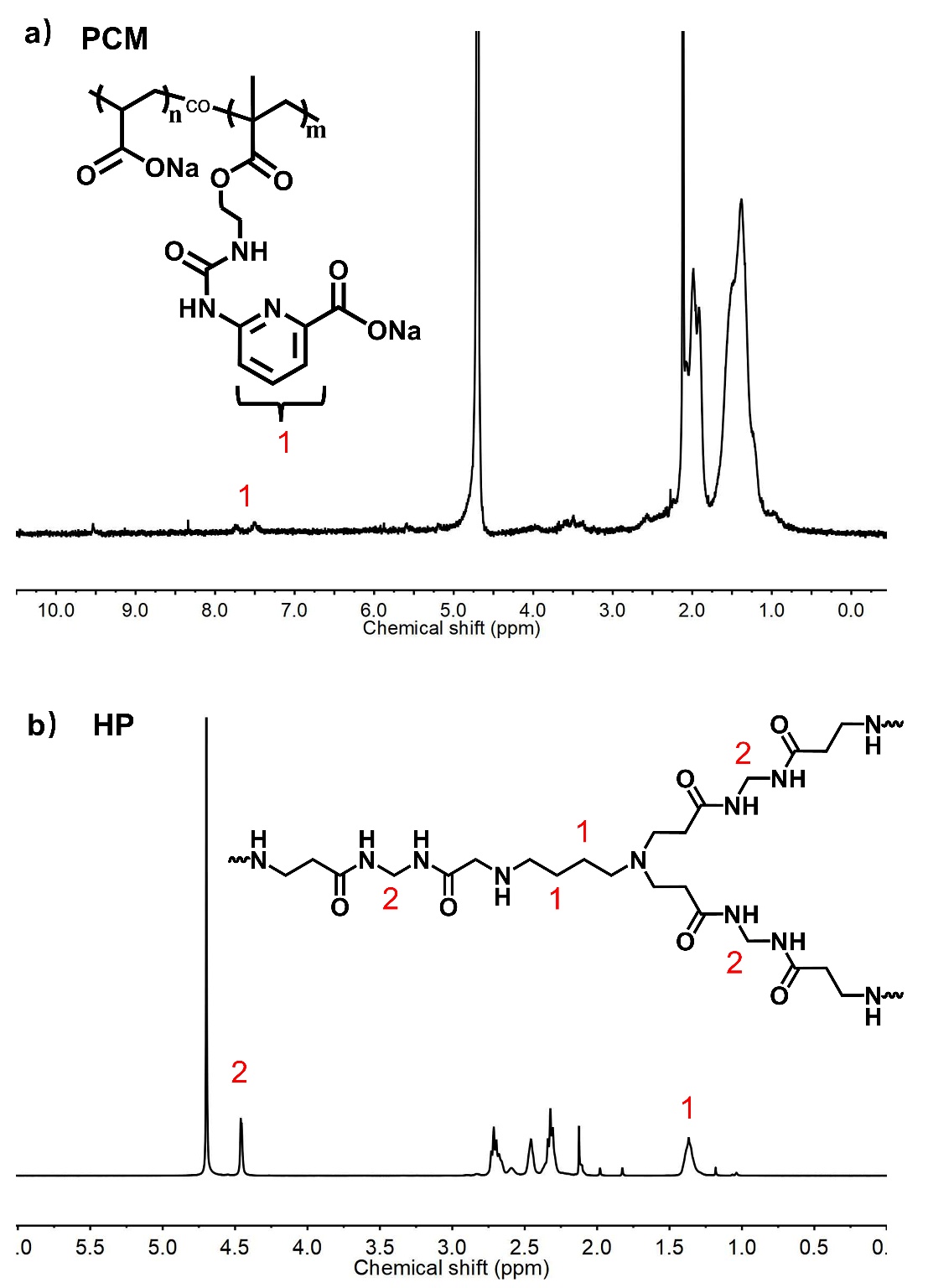


**Figure S2.** Structure characterization of polymers. (a) ^1^H NMR spectra of PCM in D_2_O. (b) ^1^H NMR spectra of HP in D_2_O.

**Figure S3.**


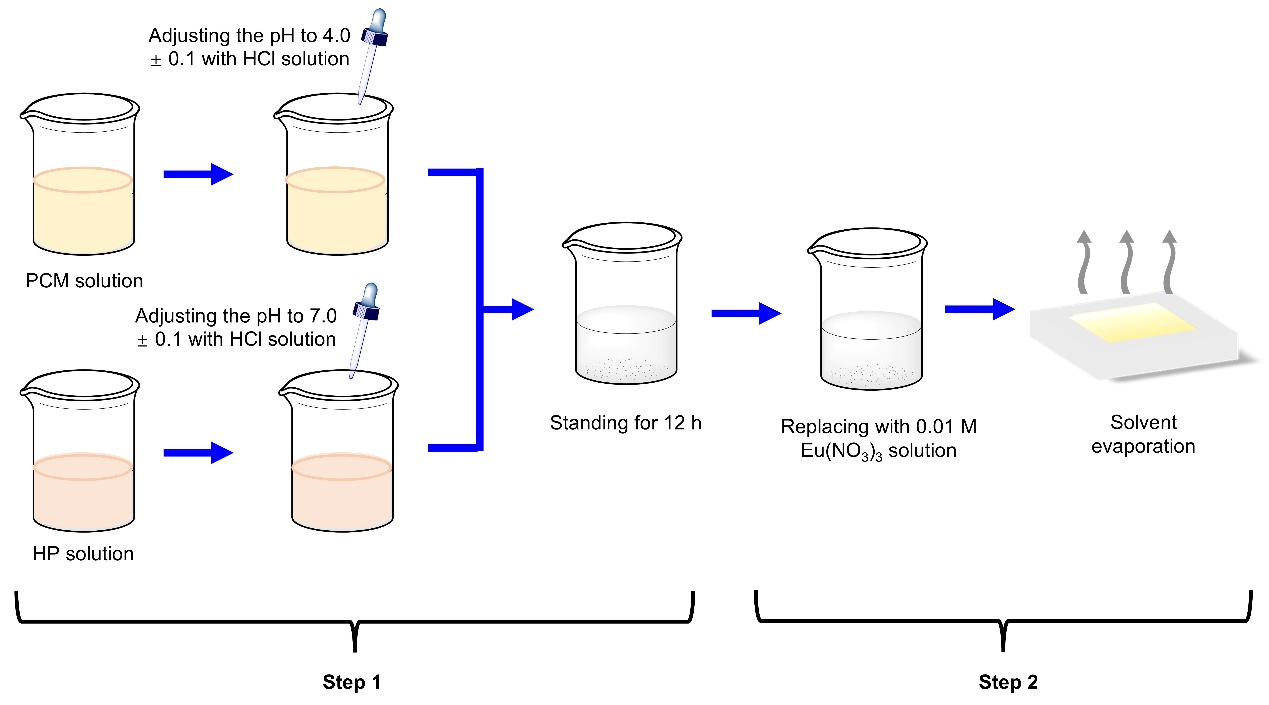


**Figure S3.** Preparation process of Eu-PCM/HP film.

**Figure S4.**


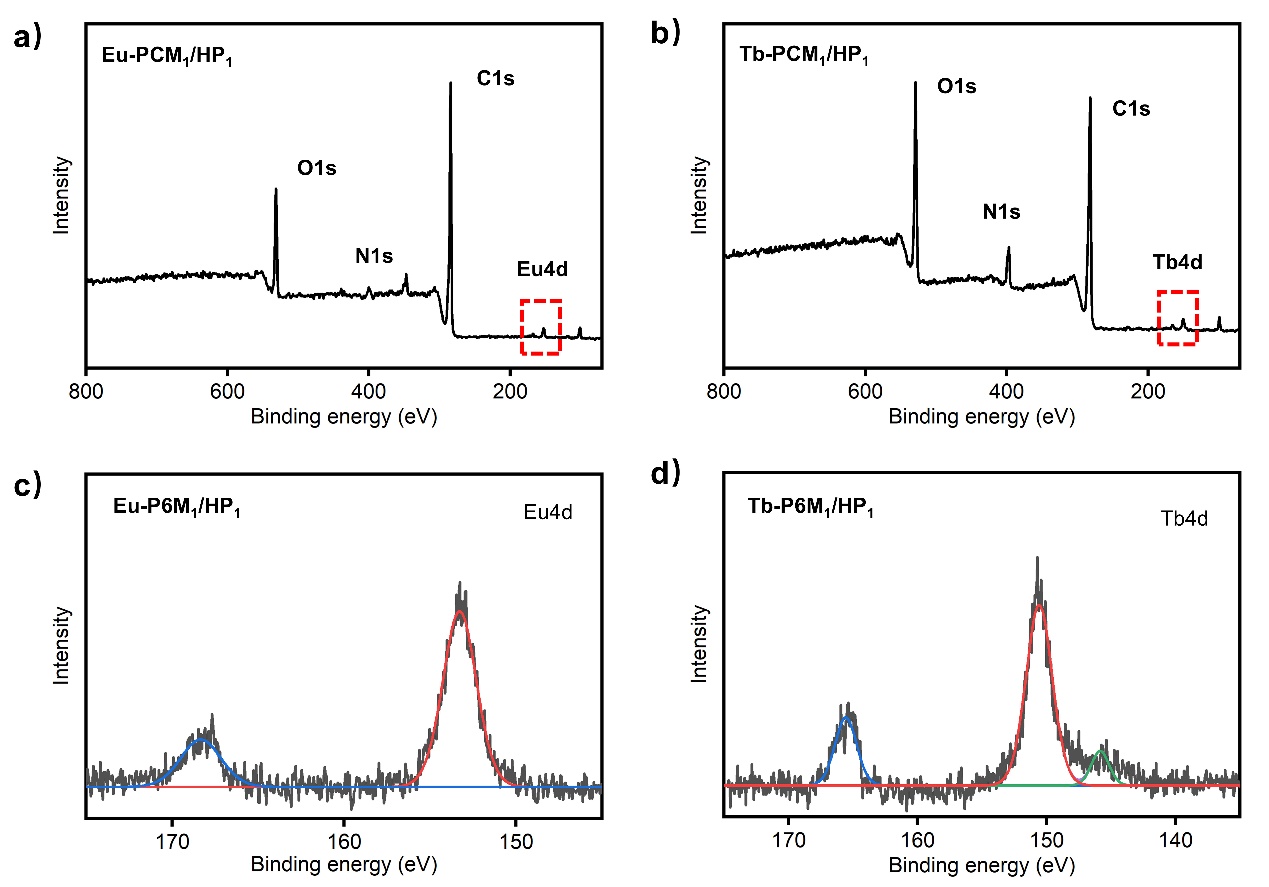


**Figure S4.** X-ray photoelectron spectroscopy (XPS) characterization. (a) XPS spectra of Eu-PCM_1_/HP_1_. (b) XPS spectra of Tb-PCM_1_/HP_1_. (c) XPS fitting results for Eu4d spectra of Eu-PCM_1_/HP_1_. (d) XPS fitting results for Tb4d spectra of Tb-PCM_1_/HP_1_.

**Figure S5.**


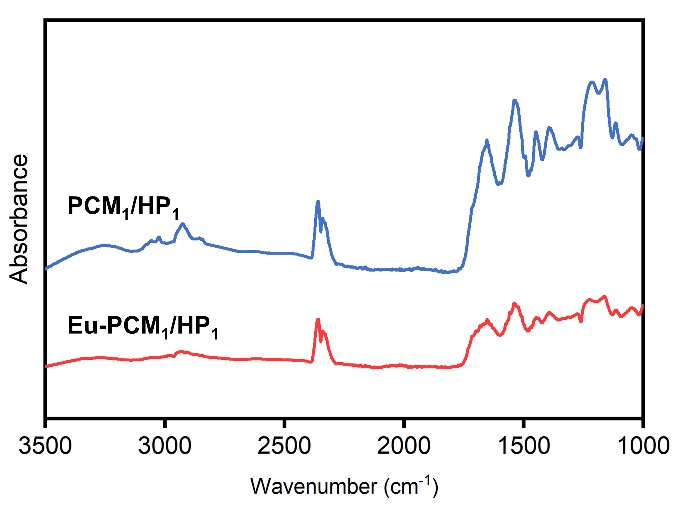


**Figure S5.** Fourier Transform Infrared (FT-IR) spectra of the PCM_1_/HP_1_ and Eu-PCM_1_/HP_1_ samples.

**Figure S6.**


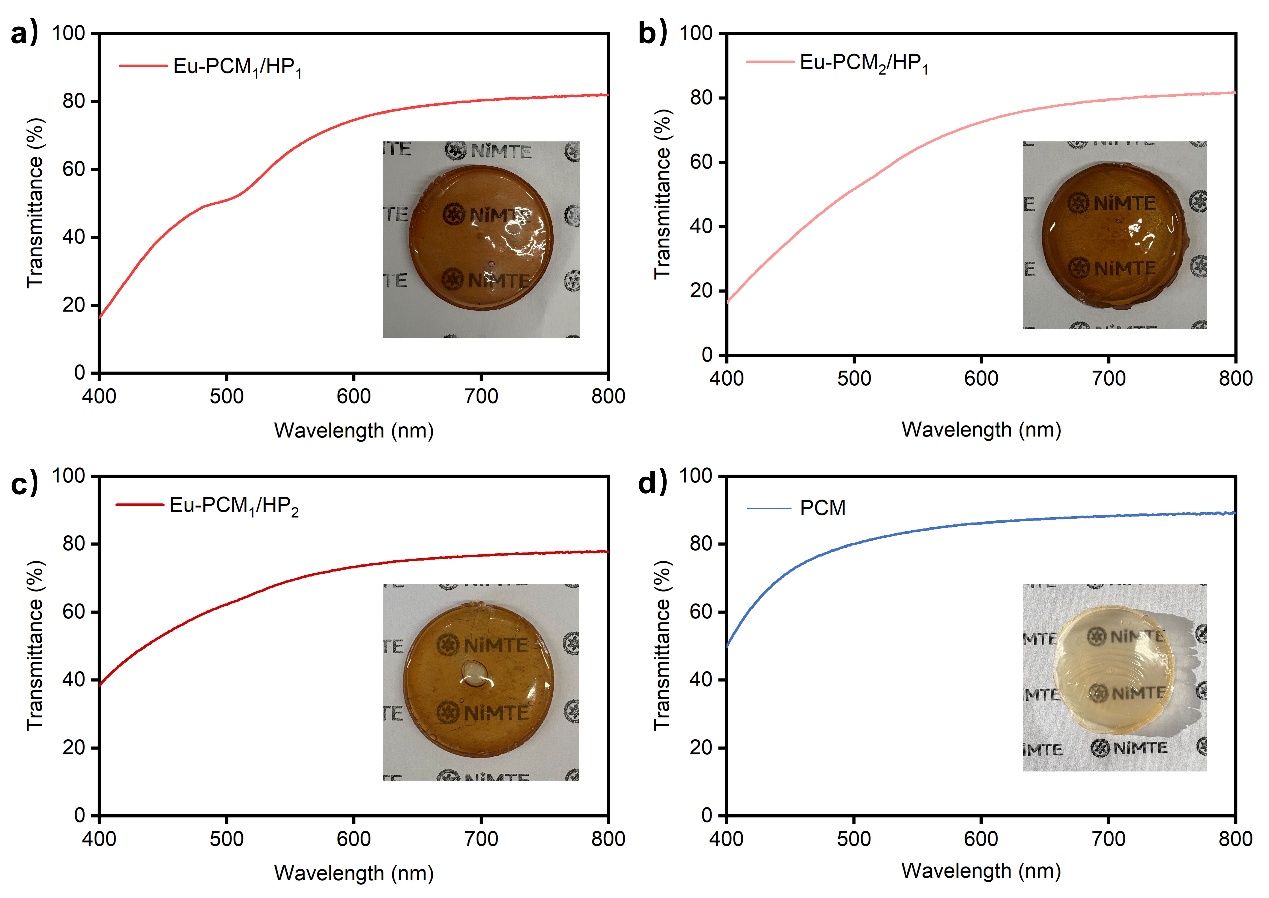


**Figure S6.** Optical transmittance of the supramolecular films. Optical transmittance of (a) Eu-PCM_1_/HP_1_, (b) Eu-PCM_2_/HP_1_, (c) Eu-PCM_1_/HP_2_ with a film thickness of 1 mm, and (d) PCM with a film thickness of 0.5 mm. The inset are photographs of the corresponding polymer films.

**Figure S7.**


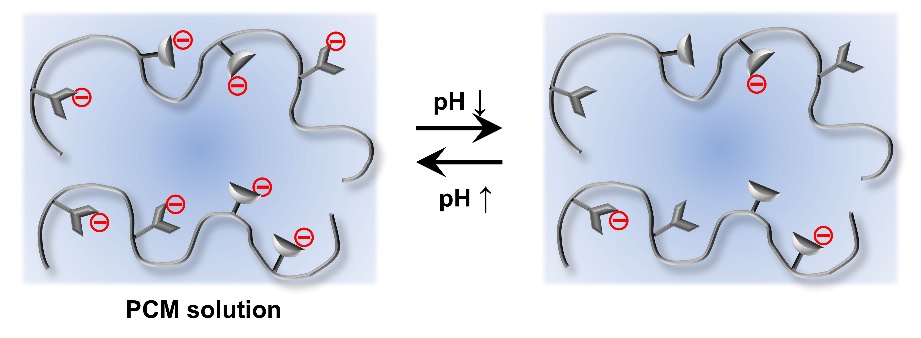


**Figure S7.** Schematic diagram of ionization of PCM solution before and after pH adjustment.

**Figure S8.**


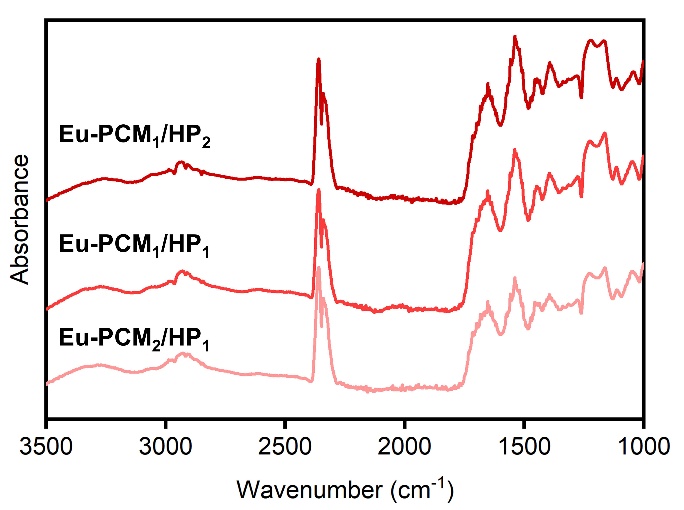


**Figure S8.** FT-IR spectra of the Eu-PCM_2_/HP_1_, Eu-PCM_1_/HP_1_, and Eu-PCM_1_/HP_2_ samples.

**Figure S9.**


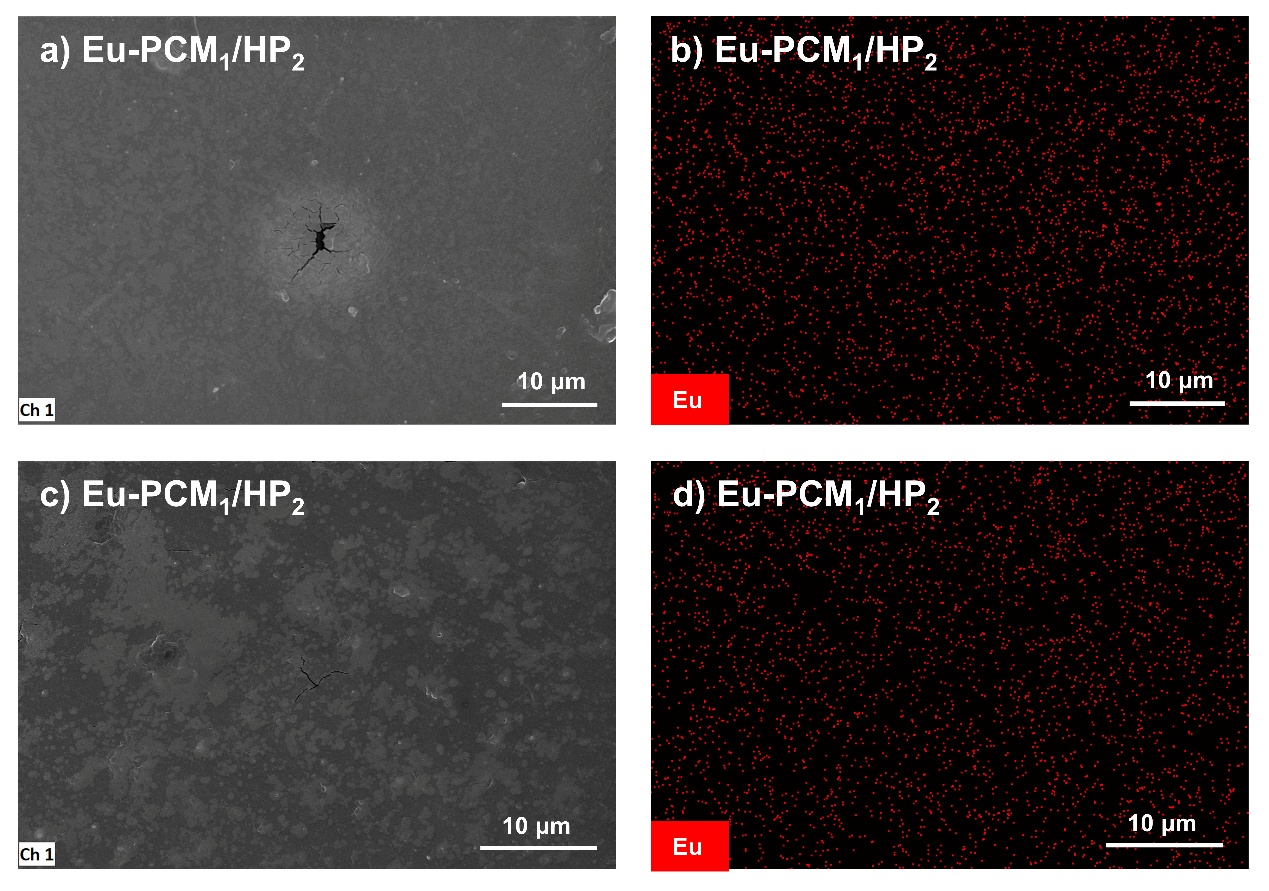


**Figure S9.** Surface microscopic morphology of supramolecular films. (a) The SEM image of Eu-PCM_1_/HP_2_ film. (b) The EDS mapping of Eu-PCM_1_/HP_2_ film. (c) The SEM image of Eu-PCM_2_/HP_1_ film. (b) The EDS mapping of Eu-PCM_2_/HP_1_ film.

**Figure S10.**


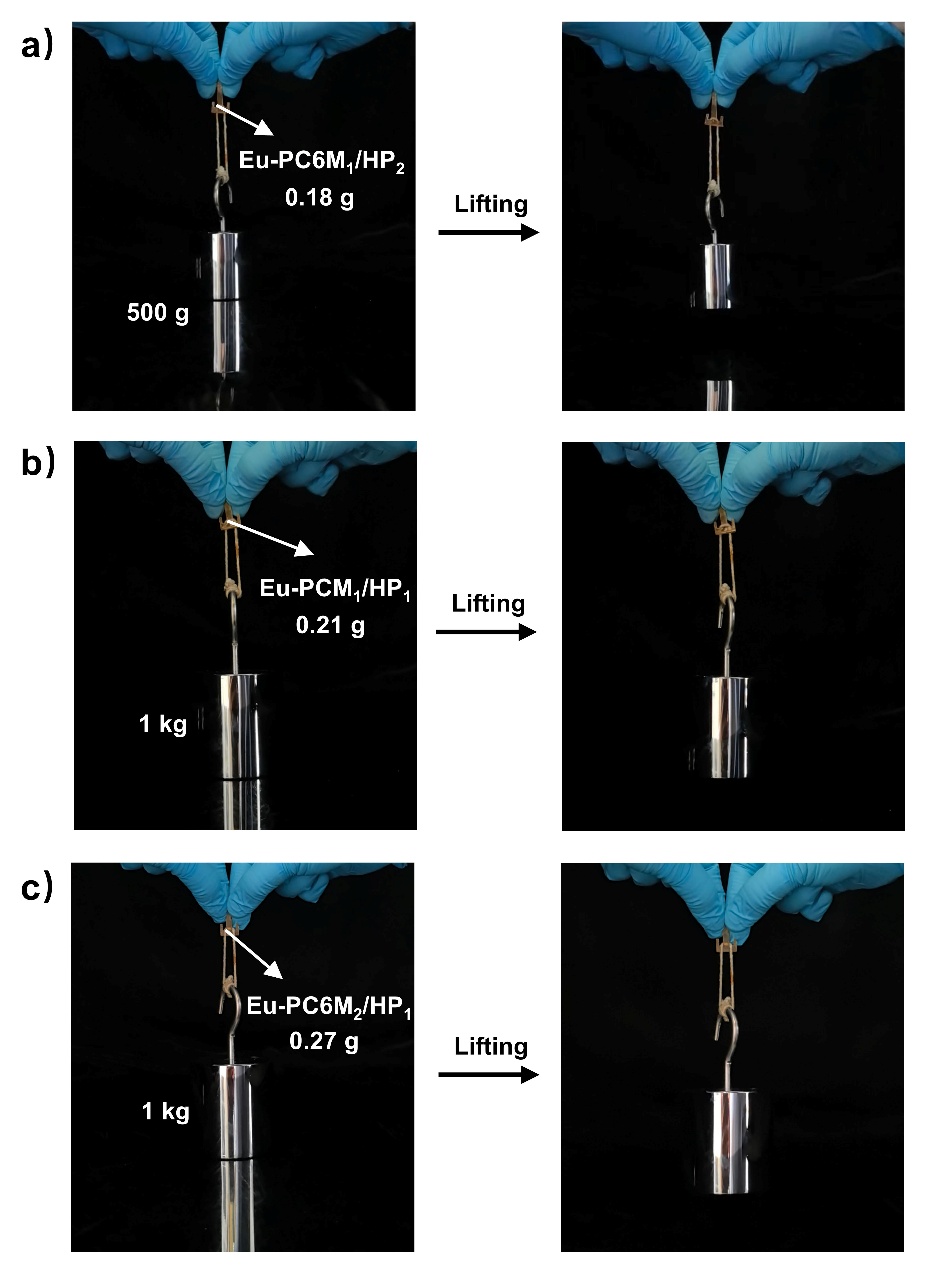


**Figure S10.** Mechanical properties of the supramolecular films. (a) The photo showing that 0.18 g of Eu-PCM_1_/HP_2_ film can lift a 500 g weight. (b) The photo showing that 0.21 g of Eu-PCM_1_/HP_1_ film can lift a 1 kg weight. (c) The photo showing that 0.27 g of Eu-PCM_2_/HP_1_ film can lift a 1 kg weight.

**Figure S11.**


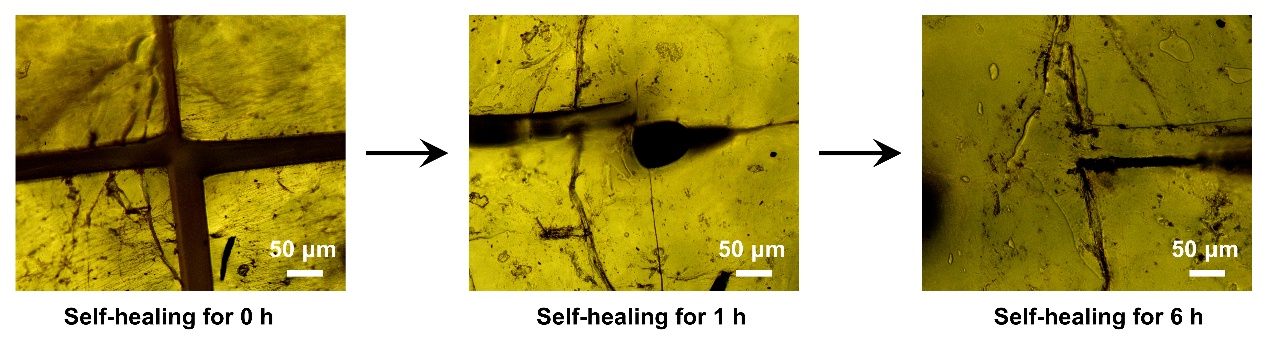


**Figure S11.** Optical microscopy images showing the self-healing process of the Eu-PCM_2_/HP_1_ film.

**Figure S12.**


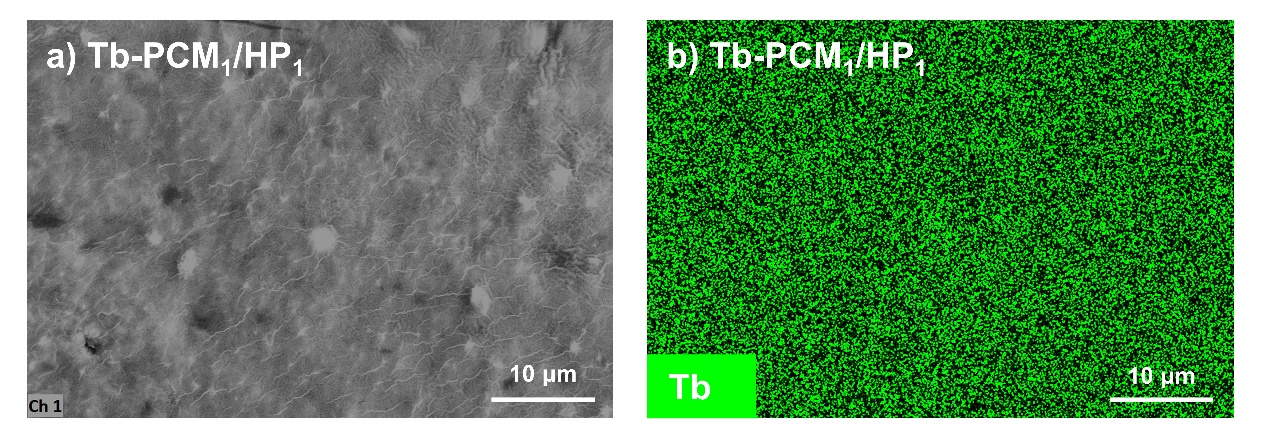


**Figure S12.** Surface microscopic morphology of supramolecular films. (a) The SEM image of Tb-PCM_1_/HP_1_ film. (b) The EDS mapping of Tb-PCM_1_/HP_1_ film.

**Figure S13.**


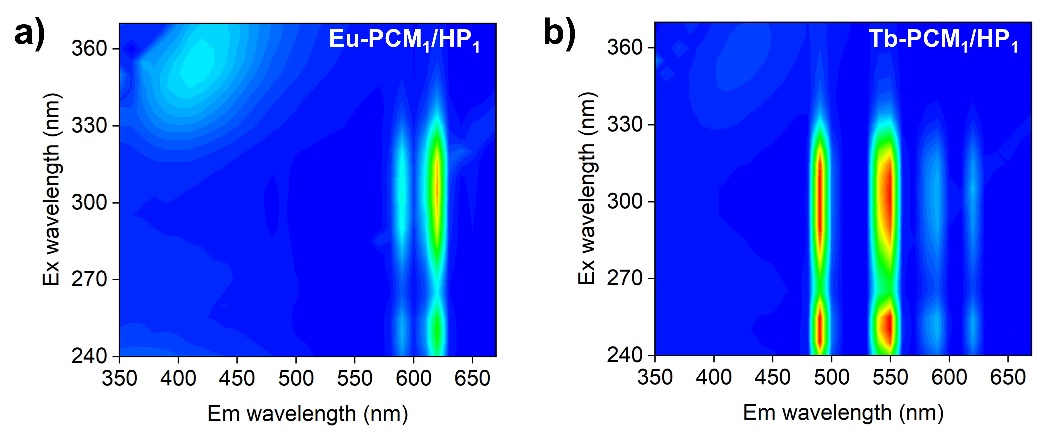


**Figure S13.** Fluorescence performance of PCM/HP films. (a) 3D fluorescence mapping of the Eu-PCM_1_/HP_1_. (b) 3D fluorescence mapping of the Tb-PCM_1_/HP_1_ films.

**Figure S14.**


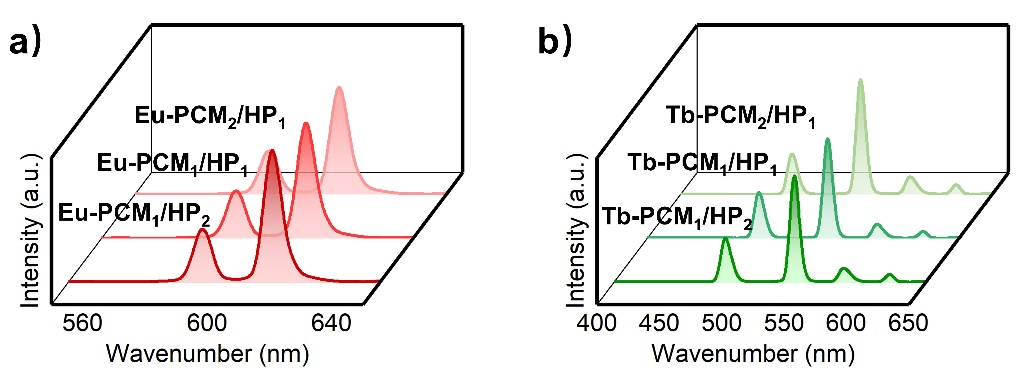


**Figure S14.** Fluorescence performance of PCM/HP films with different component ratios. (a) Fluorescence spectra of Eu-PCM_1_/HP_2_, Eu-PCM_1_/HP_1_, and Eu-PCM_2_/HP_1_. (b) Fluorescence spectra of Tb-PCM_1_/HP_2_, Tb-PCM_1_/HP_1_, and Tb-PCM_2_/HP_1_. Excitation at 254 nm for the fluorescence spectral measurements.

**Figure S15.**


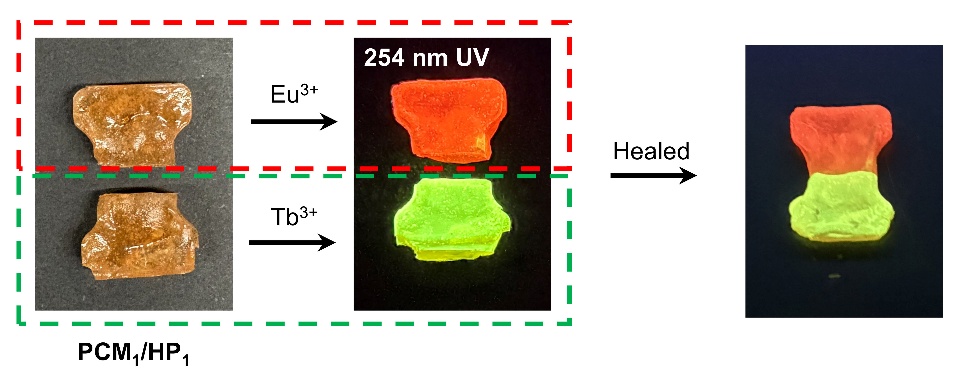


**Figure S15.** Photographs showing the self-healing process under 254 nm UV after treatment of PCM_1_/HP_1_ samples with Eu^3+^ or Tb^3+^ ions.

**References**

(1) Li, W.; Zhang, H.; Lu, W.; Zhang, Y.; Zheng, T.; Yang, G.; Chen, T. *Adv. Opt. Mater.* **2023,** *11*, 2202738.

(2) Wang, H.; Liu, H.; Cao, Z.; Li, W.; Huang, X.; Zhu, Y.; Ling, F.; Xu, H.; Wu, Q.; Peng, Y.; Yang, B.; Zhang, R.; Kessler, O.; Huang, G.; Wu, J. *Proc. Natl. Acad. Sci. U S A* **2020,** *117*, 11299-11305.
